# Generalized tuberculosis due to *Mycobacterium caprae* in a red fox phylogenetically related to livestock breakdowns

**DOI:** 10.1186/s12917-022-03454-7

**Published:** 2022-09-20

**Authors:** Bernat Pérez de Val, Claudia Perea, Josep Estruch, Carlos Solano-Manrique, Carles Riera, Albert Sanz, Enric Vidal, Roser Velarde

**Affiliations:** 1 Unitat mixta d’Investigació IRTA-UAB en Sanitat Animal, Centre de Recerca en Sanitat Animal (CReSA), Campus de la Universitat Autònoma de Barcelona (UAB), Bellaterra, Catalonia Spain; 2 IRTA, Programa de Sanitat Animal, Centre de Recerca en Sanitat Animal (CReSA), Campus de la Universitat Autònoma de Barcelona (UAB), Bellaterra, Catalonia Spain; 3 National Veterinary Services Laboratories, U.S. Department of Agriculture, Ames, IA USA; 4 Wildlife Ecology & Health group (WE&H) and Servei d’Ecopatologia de Fauna Salvatge (SEFaS), Departament de Medicina i Cirurgia Animals, Universitat Autònoma de Barcelona, Bellaterra, Catalonia Spain; 5 Centre de Fauna de Vallcalent, Generalitat de Catalunya, Lleida, Catalonia Spain; 6 Departament d’Acció Climàtica, Alimentació i Agenda Rural de la Generalitat de Catalunya, Barcelona, Catalonia Spain

**Keywords:** Tuberculosis, *Mycobacterium caprae*, Fox, Livestock, Whole genome sequencing

## Abstract

**Background:**

Tuberculosis (TB) due to *Mycobacterium caprae* is endemic in goat herds and free-ranging wild boars in Spain, causing infections in other livestock or wild animals to a lesser extent. TB infection in foxes is infrequently reported and they are usually considered spillover hosts of TB.

**Case presentation:**

A blind, depressed and severely emaciated red fox (*Vulpes vulpes*) was admitted to a rehabilitation center. After clinical examination it was humanely sacrificed. At necropsy, generalized TB lesions were observed that were subsequently confirmed by histopathology along with a co-infection with canine distemper virus. *M. caprae* was isolated from mycobacterial culture and spoligotype SB0415 was identified. Whole genome sequencing (WGS) of the isolated *M. caprae* was carried out and single nucleotide polymorphisms (SNP) were compared with other sequences of *M. caprae* isolated from livestock and wildlife of the same area throughout the last decade.

**Conclusions:**

This is the first reported case of TB due to *M. caprae* in a fox in the Iberian Peninsula. WGS and SNP analysis, together with spatial-temporal investigations, associated this case with recent *M. caprae* outbreaks in cattle and goat herds of the area. The results indicated transmission of *M. caprae* between livestock and the fox, suggesting that this species may occasionally play a role in the epidemiology of animal TB.

## Background

*Mycobacterium caprae* is the main cause of goat tuberculosis (TB) in Spain [1] and has also been involved in TB outbreaks in other livestock and wildlife species in contact with infected goats, such as cattle [2], sheep [3], and wild boars [4]. Moreover, *M. caprae* may be a source of human TB [5], mainly through inhalation of mycobacteria, but ingestion from contaminated dairy products cannot be ruled out.

The circulation of *Mycobacterium tuberculosis* complex (MTBC), mainly *M. bovis* and *M. caprae*, in non-bovine domestic and wild hosts is hampering the success of bovine TB eradication campaigns in Spain, which has been practically stagnant for the last two decades [6]. The red fox *(Vulpes vulpes)* has been usually considered a spillover host of TB and it is rarely included in the active surveillance of the disease, therefore only sporadic cases of TB due to *M. bovis* have been detected to date in Spain [7, 8].

Nowadays, the use of whole genome sequencing (WGS) for epidemiological investigation of animal TB outbreaks and to study putative transmission events is becoming more widespread. A case of red fox (*Vulpes vulpes*) with disseminated TB in Catalonia (Spain), posed the first detection of *M. caprae* in this species in the Iberian Peninsula, and the investigation of its origin by using epidemiological and WGS analysis aimed this study.

## Case presentation

In February 2021, an adult female free-ranging red fox was found blind and depressed in southern Catalonia (Spain). The animal was admitted to *Vallcalent* Wildlife Rehabilitation Centre (Lleida, Catalonia). The fox was severely emaciated (2.94 kg), with abundant ectoparasites (ticks and fleas) and filiform worms consistent with *Thelazia callipaeda* in both conjunctival sacs. After clinical examination, it was humanely euthanized. Afterwards, according to the Catalan Wildlife Health Surveillance Plan, it was submitted to the Veterinary Faculty of the Autonomous University of Barcelona (Bellaterra, Spain) to carry out a complete necropsy. Tissue samples from brain, eyes, retropharyngeal, thoracic, and mesenteric lymph nodes, heart, pericardium, trachea, lungs, liver, spleen, kidneys, urinary bladder, adrenal glands, and bone marrow were fixed in 4% neutral buffered formalin and processed and embedded in paraffin for routine microscopic examination. Intestinal samples were not collected since they were too autolytic for their precise evaluation. Sections of 3–4 μm thick were stained with Mayer’s haematoxylin and eosin. Ziehl-Neelsen staining was also carried out to detect acid-fast bacilli. Immunohistochemistry was used to evaluate the presence of canine distemper virus (CDV) antigen in selected sections from the brain and lung. A monoclonal antibody against the nucleoprotein of CDV was used (from VMRD, Ref.: CDV-NP, Ascites fluid, 1 mg/mL, IgG2b, kappa light chain, dilution used 1:5000).

Fresh samples of kidney and mediastinal lymph node with TB compatible lesions were processed for bacteriology. Tissues were mechanically homogenized in 10 ml of sterile water using an automatic homogenizer (Masticator, IUL, Barcelona, Spain), decontaminated with 10 ml of 5% w/v oxalic acid for 30 min. in orbital shaking, neutralized with 5 ml of sodium hydroxide 1 M and centrifuged at 2471×g for 30 min. Supernatants were discarded, pellets were resuspended with 1 ml of sterile phosphate buffered saline, and an aliquot of 0.5 ml was inoculated in BBL MGIT supplemented tubes and incubated in the BACTEC MGIT 320 system (BD Diagnostics, Sparks, MD, USA). The remaining 0.5 ml was cultured using swabs in Löwenstein-Jensen with pyruvate and Coletsos solid media (BD Diagnostics,) and incubated at 37 °C.

Culture growth was confirmed as MTBC by multiplex PCR [9]. DNA from MTBC growth was molecularly characterized by DVR-spoligotyping, at VISAVET Health Surveillance Centre (Madrid, Spain), and WGS followed by Single Nucleotide Polymorphisms (SNPs), at the National Veterinary Services Laboratories of the US Department of Agriculture (Ames, IA, USA).

**Fig. 1:**
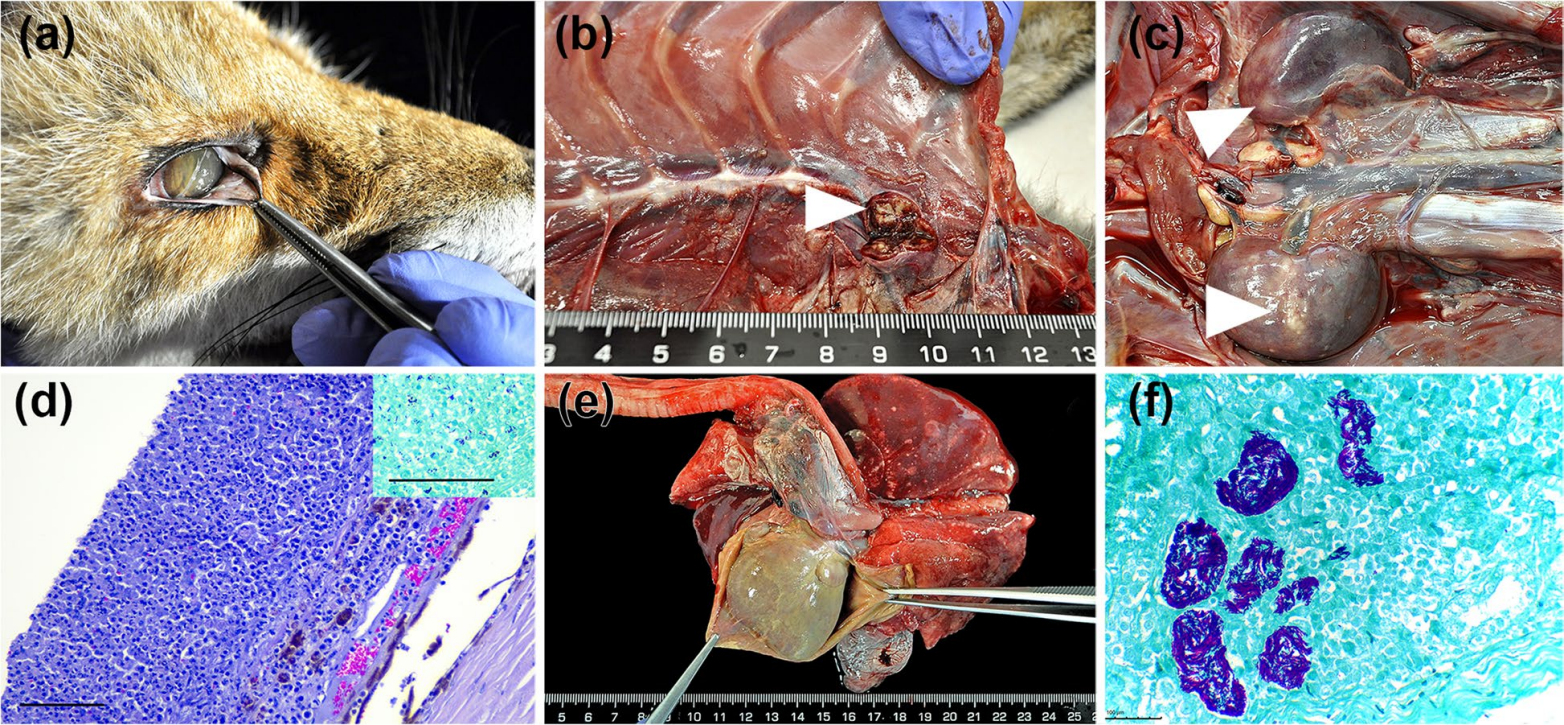
Red fox with generalized tuberculosis and canine distemper virus coinfection. Gross and histopathological findings. **a** Right eye, mild corneal edema and hypopyon, and few filiform worms consistent with *Thelazia callipaeda* in the conjunctival sac. **b** Cranial mediastinal lymph node with multifocal yellow areas of caseous necrosis. **c** Multiple foci of caseous-purulent necrosis in both kidneys. **d** Right eye, dense perivascular to diffuse infiltration of the choroid with histiocytes and lymphocytes, and a dense subretinal pyogranulomatous exudate (Hematoxylin and eosin stain, original magnification × 200, bar = 100 μm), Inset, pyogranulomatous exudate with multiple purple-stained acid-alcohol resistant bacteria (Ziehl-Neelsen stain, original magnification × 200, bar = 100 μm). **e** Diffuse thickening of the pericardium and abundant caseous-purulent exudate filling the pericardial cavity. **f** High quantity of acid-alcohol resistant bacteria within macrophages and necrotic areas in the kidneys (Ziehl-Neelsen stain, original magnification × 400, bar = 100 μm). All images taken by the authors

At necropsy, the fox was severely emaciated. In the eyes, diffuse corneal edema, hypopyon, and the presence of a few filiform worms in the conjunctival sac, consistent with *Thelazia callipaeda*, were noted (Fig.1a). Internally, retropharyngeal, prescapular, and mesenteric lymph nodes were moderately enlarged, and multifocal yellow areas of caseous necrosis were seen in the cranial mediastinal lymph node (Fig.1b). There was a diffuse severe thickening of the pericardium and abundant caseopurulent exudate filled the pericardial cavity (Fig.1e). In the heart, white mild nodular thickening of the left atrio-ventricular and aortic valves was detected. From these affected valves, the inflammation extended locally into the myocardium. In the lungs, an increased consistency and brown-gray discoloration of the caudo-dorsal areas, consistent with verminous pneumonia, was recognized. Several discrete white granulomas, 2 to 4 mm in diameter, were noted in the left parietal pleural surface. The liver was pale orange, had slightly rounded margins, and subtle multifocal to coalescent inflammatory infiltrates. A diffuse, mild splenomegaly was also present. Severe multifocal to coalescent foci of caseous-purulent necrosis were found in both kidneys (Fig. 1c). A moderate number of cestodes was seen in the small intestine.

Microscopically, diffuse granulomatous infiltrates and/or granulomas compatible with TB lesions were seen in most tissues. Diffuse granulomatous infiltrates were present in the pericardium, epicardium, heart valves, interstitial renal medulla, and choroid layer in the eyes. Discrete miliary (up to 200 μm), round to oval granulomas, characterized by a central core of macrophages surrounded by a thin rim of lymphocytes and plasma cells were numerous in the cortical area of the kidneys, liver, lymph nodes and spleen. A few small granulomas were also found in the brain, bone marrow and lung. None of the granulomas were encapsulated. The number of multinucleated giant cells was low and most of them had only two to three nuclei (small). Areas of caseous necrosis containing few degenerate neutrophils were present in both granulomatous infiltrates and granulomas. Small foci of mineralization were only seen in the valves. The pericardial sac and epicardium were markedly thickened with fibroblasts proliferation and fibrosis, suggesting that this was the oldest lesion.

In the eyes, a severe granulomatous choroiditis and vitritis were established as the cause of the blindness (Fig.1d). The exudate in the vitreous chamber caused the detachment and degeneration of the retina in both eyes. In the right eye, the granulomatous infiltrate and exudate extended to the anterior uvea and chamber explaining the hypopyon. A mild keratitis characterized by mild superficial edema and neutrophilic infiltrate with incipient neovascularization at the limbus was also present. No inclusion bodies were seen in the corneal or conjunctival epithelium.

Numerous acid-fast and rod-shaped bacilli were seen within the macrophages and necrotic areas in all tissues, including foamy macrophages in the lumen of the bronchi and renal pelvis consistent with an active shedding of mycobacteria (Fig.1f).

Additionally, lesions consistent with CDV co-infection were also present in the brain and the lungs. Mild perivascular lymphocytic cuffing was noted in the section from the hippocampus in the brain. In the lung, the presence of patchy necrosis, and attenuation of bronchiolar epithelium along with lymphocytes around bronchioles and vessels, alveolar edema, and the occasional alveolar epithelial syncytial cell were consistent with multifocal viral subacute bronchiolo-interstitial pneumonia. Canine distemper virus antigen was detected in bronchiolar epithelial cells, alveolar macrophages in the lung and in the occasional neuron, glial, and ependymal cell in the brain.

Verminous pneumonia was also confirmed. Arterial damage within the lesions suggested *Angiostrongylus vasorum* as the possible etiology, although no adult parasites were present in the sections. The main final diagnosis was a generalized TB infection with a co-infection with canine distemper virus.

Culture results revealed growth of *M. tuberculosis* complex (MTBC) in both tissues, and the spoligopattern *M. caprae* SB0415 (www.mbovis.org) was identified. Then, the whole genome sequences were analyzed together with other 9 recent *M. caprae* SB0415 isolates (2018-2021) from cattle (1), goat (1) and wild boar (7) of the same area and other 17 available whole genome sequences of previous *M. caprae* isolates in Catalonia (2008-2020) [4].

**Fig. 2:**
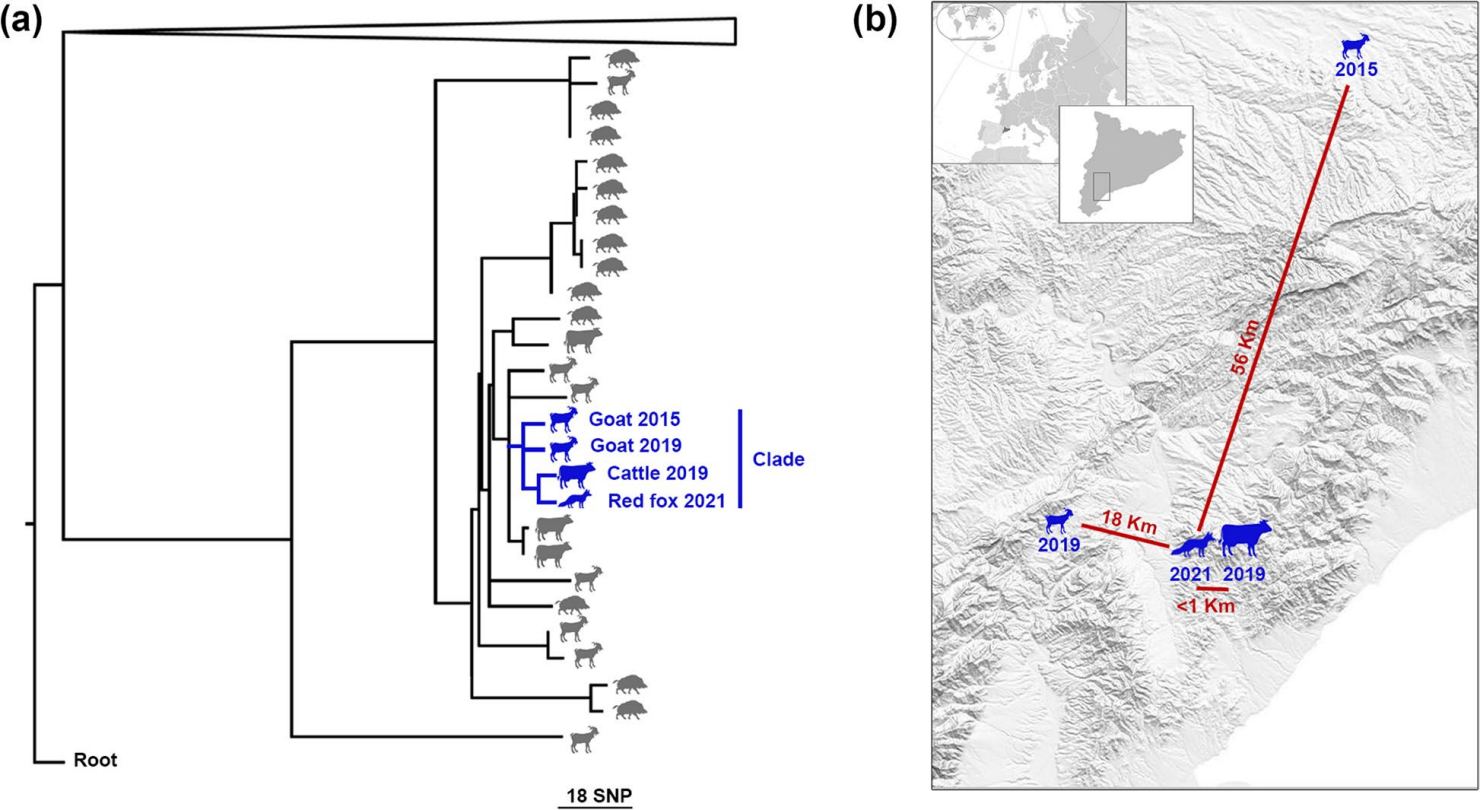
Phylogenetic and spatiotemporal relationships of *M. caprae* strains. **a** Phylogenetic tree of *M. caprae* isolates in Catalonia. A rooted phylogenetic tree, based on maximum likelihood model (GTR-CAT, RAxML), shows the SNP distance of the 27 *M. caprae* strains isolated in Catalonia between 2008 and 2021. Infected animal species (cattle, goats, wild boars or fox) are shown with silhouettes next to each branch. The strain isolated from the fox (blue silhouette) formed a clade together with the strain isolated from a cattle herd (blue silhouette, < 1Km) and two goat herds (blue silhouettes, 18 and 56 Km, respectively). The dates of isolation are shown next to the silhouettes. **b** Location of the *M. caprae* clade isolates. The inset shows a region of southwestern Catalonia. Species are shown by silhouettes. Dates of isolation and distance between the fox and livestock herds are indicated in the map

WGS analysis showed a close phylogenetic relationship between the fox isolate and another one (8 SNP pairwise distance) isolated 1 year before from a cattle herd located at the same municipality where the ill fox was found (Fig. 2). In addition, the strains isolated from two goat herds in 2015 and 2019, located at 56 and 18 Km from the cattle-fox outbreak, respectively, also showed a close phylogenetical relationship with the fox strain (9 SNP pairwise distance, Fig. 2). These four isolates share a common ancestor within 6.5 SNP (range 5-8).

Additionally, in the context of the Catalan wildlife health surveillance plan, 3 out of the 9 (33%) wild boar sera sampled in the outbreak municipality in 2020 were seropositive to TB by ELISA [10], while all wild boar sera obtained before the cattle breakdown (18, between 2015 and 2019) and after the detection of the diseased fox (3 in 2021) were seronegative. Even though no wild boar tissues were sampled to confirm the infection, these results suggest that mycobacteria could be circulating among wild boars after the cattle outbreak.

## Discussion and conclusions

The involvement of the red fox in the epidemiology of animal TB remains largely unknown. However, cases of TB in foxes due to *M. bovis* have been increasingly reported in European endemic areas of bovine TB [7, 8, 11–14]. There is limited evidence for cattle-to-fox transmissions and vice versa [13]. Here, we demonstrated close spatiotemporal and phylogenetic relationships that suggest direct or indirect transmission of *M. caprae* between both species. In addition, at least two goat herds might be related with the origin of this outbreak according to SNP pairwise distance, estimated in < 12 SNP distance between individuals in other studies [4, 15]. Unfortunately, only fragmentated data on goat TB cases is available due to the absence of a mandatory caprine TB eradication program, thus limiting a more accurate understanding of the outbreak.

In Southern Catalonia, there is a predominance of livestock herds with extensive management, sometimes with limited biosecurity, thus facilitating the interaction between wildlife and livestock. Maintenance of TB within multi-host communities has been identified in some European regions [16]. Indeed, in the epidemiological context of the outbreak region, cattle, goats and wild boars may constitute a multi-host maintenance community of both *M. bovis* and *M. caprae,* with particular emphasis on goat herds which are not yet subjected to an official eradication program [10, 17]. Even though an infected cattle herd could be the most likely source of infection for the fox, the participation of wild boars as intermediate hosts cannot be ruled out since seropositive animals were detected in the same municipality between the detection of the infection in the cattle herd and the fox.

*M. caprae* infection in wildlife has been recently reported in Catalonia [4] as well as in Central and Eastern European regions [18, 19]. This study further supports the evidence that foxes are susceptible to *M. caprae* infection that may evolve to disseminated TB. A case of generalized TB due to *M. caprae* was recently reported in Austria [20], where *M. caprae* is the main causative agent of TB in cattle and red deer [21]. To our knowledge, we report here the first case of TB due to *M. caprae* in a fox in the Iberian Peninsula.

Blindness caused by ocular TB is a rare presentation of the disease even in domestic animals. Ocular infection is usually the result of the spread of the bacteria from the primary site to the eye via the hematogenous route, frequently reaching the eye through the choroid [22] as in this case. Diffuse tuberculous pericarditis is also an uncommon presentation. This lesion is more often caused by the spread of the bacteria from the mediastinal lymph nodes directly into the pericardium than from tuberculous pneumonia [23]. The pericarditis with fibrous thickening was the oldest lesions seen in this animal. The co-infection with canine distemper virus, an immunosuppressive virus that can probably influence the TB outcome in foxes [20], could have facilitated the sudden dissemination of the infection. Viral inclusions were not readily seen which was consistent with a later stage of the disease. Co-infections, such as the conspicuous parasitism observed in this animal, may have had also an influence on the generalized presentation of TB in this case [24].

While in some cases MTBC infection does not induce TB visible lesions in foxes [13], in this report, in consistency with others, it evolves to generalization [8, 16]. Interestingly, even in lower pathogenic forms, mycobacterial shedding has been detected in feces, urine and oropharyngeal mucus [12, 13], and this excretion can be increased in generalized forms. Indeed, lesions in the kidneys of this case had remarkable amounts of mycobcateria (Fig. 1), suggestive of extensive bacterial shedding through urine.

Therefore, our results further evidence that foxes may act as TB spillovers in endemic areas but also might be an indirect source of infection for livestock and other wildlife species under suitable ecological conditions. Also, the risk for human health should not be ignored, especially when these animals are clinically evaluated and handled in a wildlife center or manipulated by hunters/furriers. This report highlights the importance of considering TB in the differential diagnosis of emaciated foxes. Including this species in wildlife TB surveillance campaigns is strongly recommended.

## Data Availability

The datasets used and/or analysed during the current study are available from the corresponding author on reasonable request.

## Abbreviations

TB: Tuberculosis
MTBC: *Mycobacterium tuberculosis* complex
WGS: Whole Genome Sequencing
SNP: Single Nucleotide Polymorphisms
